# Serological biomarkers predict immune-related adverse events and clinical benefit in patients with advanced gastrointestinal cancers

**DOI:** 10.3389/fimmu.2022.987568

**Published:** 2022-09-08

**Authors:** Yanni Wang, Jianling Zou, Yun Li, Xi Jiao, Yujiao Wang, Na Zhuo, Mengting Gao, Jifang Gong, Jian Li, Xiaotian Zhang, Xicheng Wang, Zhi Peng, Changsong Qi, Zhenghang Wang, Jie Li, Yan Li, Lin Shen, Henghui Zhang, Zhihao Lu

**Affiliations:** ^1^ Department of Gastrointestinal Oncology, Key Laboratory of Carcinogenesis and Translational Research (Ministry of Education/Beijing), Peking University Cancer Hospital and Institute, Beijing, China; ^2^ Department of Medical Affairs, Genecast Precision Medicine Technology Institute, Beijing, China; ^3^ Institute of Infectious Diseases, Beijing Ditan Hospital, Capital Medical University, Beijing, China

**Keywords:** gastrointestinal cancers, immune checkpoint inhibitors, immune-related adverse events, biomarker, cytokines

## Abstract

**Background:**

Immune checkpoint inhibitors (ICIs) have dramatically improved survival in advanced gastrointestinal (GI) cancer patients, but also resulted in immune-related adverse events (irAEs). This study aimed to evaluate serological biomarkers of irAEs and treatment response in GI cancer patients.

**Patients and methods:**

Metastatic GI cancer patients were enrolled between August 1, 2015, and July 31, 2017. Serum samples were collected at baseline, and a panel of 59 serum biomarkers was tested. The occurrence of irAEs was analyzed, and serological biomarker expression was correlated with irAE incidence and prognosis.

**Results:**

Fifty-one patients were enrolled, of whom 47.1% (24/51) were diagnosed with irAEs, including 4 patients (7.8%) with grade 3-5 irAEs. The most common irAE was thyroiditis (9/51, 17.6%), followed by colitis (7/51, 13.7%). The expression of CD28 (P = 0.042), IL-4 (P = 0.033), IL-15 (P = 0.024) and PD-L1 (P = 0.018) was significantly elevated in patients with grade 3-5 irAEs. For organ-specific irAEs, IL-6 levels were higher in patients with thyroiditis and colitis, while IL-22 and SCF levels were higher in patients with colitis. Increased IL-1α, IL-21, LIF, and PIGF-1 levels were significantly associated with myositis incidence, while the serum levels of six cytokines (BTLA, GM-CSF, IL-4, PD-1, PD-L1 and TIM-3) were higher in patients with rash. Prognostic analysis showed that patients with irAEs had better tumor response (P = 0.029), improved PFS (median survival: undefined vs. 2.1 months, P = 0.002), and extended OS (median survival: undefined vs. 4.3 months, P = 0.003). The prognostic value of irAEs was only significant in patients who received anti-PD-1 inhibitors, but not in those who received anti-PD-L1 inhibitors. Besides, elevated BTLA (median OS: not reached vs. 7 months; P = 0.0168) and PD-1 (median OS: not reached vs. 7 months; P = 0.0223) concentrations were associated with longer OS.

**Conclusions:**

Serological proteins are promising markers for predicting immune-related toxicity and prognosis in GI cancer patients. Organ-specific irAEs have various cytokine profiles. Although further validation is needed before clinical application, this study provided a direction for identifying patients at risk for irAEs, and guiding patient selection for ICI therapy.

## Introduction

Gastrointestinal (GI) cancers are characterized by high morbidity and mortality rates (1), with limited treatment options. Systemic therapy remains the most critical treatment component for patients with local advanced or metastatic disease. However, strategies to prevent metastasis and prolong survival are urgently needed. The last 5 years have seen remarkable advances in the treatment of patients with advanced GI cancer, with the introduction of immune checkpoint inhibitors (ICIs). ICIs can produce durable anti-tumor immune responses and increase overall survival (OS) in patients with GI cancers, especially microsatellite instability-high (MSI-H)/deficient DNA mismatch repair (dMMR) esophagus cancer and colorectal cancer (2, 3).

However, the enhanced immune system activity associated with ICIs leads to a unique autoimmune toxicity, known as immune-related adverse events (irAEs), defined broadly as immune-mediated host organ dysfunction secondary to aberrant immune system activity after immunotherapy (4). Excessive immunity affects multiple organ systems, including skin, liver, GI tract, and endocrine glands (5, 6). Most irAEs are grade 1-2, which are controllable. The incidence of adverse events is 10-34%, which not only negatively influences the quality of life but also interrupts treatment procedures (7). Therefore, it is important to find biomarkers for early prediction of irAEs. IrAEs may be classified as organ-specific and general irAEs. Studies have demonstrated that general adverse events are more common, but organ-specific irAEs are more clinically important and are associated with greater mortality (8). Therefore, it is essential for clinicians to focus on organ-specific irAEs, and to evaluate the benefits and risks of ICIs during cancer treatment.

A large number of serological markers may represent the immunological phenomena occurring in the tumor microenvironment, where immune and malignant cells interact (9). These might be used as predictive markers for treatment response and/or irAEs (4, 9). Some studies have explored the role of soluble cytokines in predicting irAEs. It has been reported that the combination of baseline serum TGFβ and IL-10 levels correlated with clinical outcomes, and elevated baseline IL-17 levels predicted severe diarrhea/colitis in melanoma patients after neoadjuvant ipilimumab administration (10). The CYTOX score, consisting of 11 circulating cytokines, such as IL-1a, IL-2, and IFNα2, could help in the early identification of severe immune-related toxicity (11). It was noted that the irAE patterns in different tumor types vary greatly, even with the use of the same ICIs (7). Studies on irAEs in GI cancers are limited, and the role of circulating cytokines in predicting immune-related toxicity remains unexplored.

Therefore, to identify biomarkers, to predict irAEs in metastatic GI cancers, we analyzed the expression of 59 serological markers and explored relationships between the baseline levels of serological markers and irAEs.

## Materials and methods

### Patients and study design

GI cancer patients treated with ICIs at Peking University Cancer Hospital between August 1, 2015, and July 31, 2017, were enrolled in this study retrospectively. Patients were eligible for inclusion if they had received at least 1 cycle of any ICI, including anti-PD-1 or anti-PD-L1 monotherapy, as previously described (12). Serum samples were collected at baseline. Informed consent was obtained from all patients for sample collection and further testing. The study was approved by the medical ethics committee of Peking University Cancer Hospital, and was conducted in accordance with the Declaration of Helsinki or comparable ethical standards (13).

Tumor response was assessed by medical imaging based on the modified Response Evaluation Criteria in Solid Tumors [RECIST] version 1.1 (14). The best therapeutic responses were classified, in order, as complete response (CR), partial response (PR), stable disease (SD), or progressive disease (PD), assessed from the first day of treatment to progression, death, or the last follow-up. Adverse events (AEs) were graded according to the National Cancer Institute Common Terminology Criteria for Adverse Events version 5.0. IrAEs were defined as AEs possibly related to immune dysregulation, and requiring frequent monitoring or specific treatment with immune suppression and/or endocrine replacement therapy (15).

### Serum samples and cytokine assay

Blood samples were collected by venipuncture from 51 patients at baseline, and centrifuged at 1,000 g for 10 min at 4°C. Serum was isolated and aliquoted. Sub-packages were then stored at −80°C. Cytokines were quantified using cytokine multiplex assays and enzyme-linked immunosorbent assay. A panel of 59 serum biomarkers were measured using a previously published method (12).

### Statistical analysis

Quantitative variables were presented as medians and the interquartile range (IQR). Differences between two subgroups of non-normally distributed quantitative variables were determined using the Mann-Whitney U-test. P < 0.05 was considered significant for two-sided tests. R package “survival” and “survminer” were used to identify an optimal cutoff value on the marker score. The Kaplan-Meier estimator and log-rank test were performed to analyze overall survival (OS) and progression-free survival (PFS). Statistical analyses were performed using SPSS Statistics 23.0 (IBM Corp., Armonk, NY, USA) and R version 4.1.2 (The R foundation, Vienna, Austria).

## Results

### Baseline patient characteristics and adverse events

A total of 51 GI cancer patients who received ICIs were enrolled (**Table 1**), including 66.7% (34/51) males and 33.3% (17/51) females. The median age was 52 years (range: 22-77). There were 23.5% (12/51) esophageal squamous cancers, 33.3% (17/51) gastric cancers, 21.6% (11/51) colon cancers, and 21.6% (11/51) other cancers, including liver and pancreatobiliary cancers. Among these, 64.7% (33/51) were treated with anti-PD-1 inhibitors, whereas 35.3% (18/51) were treated with anti-PD-L1 inhibitors. Outcomes included 25.5% (13/51) cases of CR or PR, 21.6% (11/51) SD, and 52.9% (27/51) PD. The characteristics of irAEs are presented in **Table 2**. In total, 47.1% (24/51) of patients were diagnosed with irAEs, including 4 (7.8%) with grade 3-5 irAEs. The most common irAE was thyroiditis (9/51, 17.6%), followed by colitis (7/51, 13.7%), presenting as diarrhea. Myositis and hepatitis were observed in 9.8% (5/51) of cases each. Moreover, 5.9% (3/51) of patients developed a rash.

**Table 1:** Patient’s characteristics.

Characteristics	All patients (N = 51)	Patients with irAEs (N = 24)	Patients with no irAEs (N = 27)
**Age at diagnosis(year)**			
Median age (range)	52 (22-77)	54 (26-77)	52 (22-71)
**Gender, N (%)**			
Male	34 (66.7)	16 (66.7)	18 (66.7)
Female	17 (33.3)	8 (33.3)	9 (33.3)
**Original site, N (%)**			
Esophagus	12 (23.5)	6 (25.0)	6 (22.2)
Stomach	17 (33.3)	8 (33.3)	9 (33.3)
Colorectum	11 (21.6)	5 (20.8)	6 (22.2)
Others	11 (21.6)	5 (20.8)	6 (22.2)
**Treatment option, N (%)**			
anti-PD-1	33 (64.7)	18 (75.0)	15 (55.5)
anti-PD-L1	18 (35.3)	6 (25.0)	12 (44.5)
**Best overall tumor response, N (%)**			
Partial Response	13 (25.5)	9 (37.5)	4 (14.8)
Stable Disease	11 (21.6)	7 (29.2)	4 (14.8)
Progressive Disease	27 (52.9)	8 (33.3)	19 (70.4)
**Response group, N (%)**			
Durable clinical benefit (DCB)	19 (37.3)	12 (50.0)	7 (25.9)
No durable benefit (NDB)	32 (62.7)	12 (50.0)	20 (74.1)

**Table 2:** Immune-related adverse events.

irAE Category	No. of patients	
	Any grade, N (%)	≥ Grade3, N (%)
**Any irAE**	24 (47.1)	4 (7.8)
**Thyroiditis**	9 (17.6)	0 (0.0)
**Colitis**	7 (13.7)	0 (0.0)
**Myositis**	5 (9.8)	1 (2.0)
**Hepatitis**	5 (9.8)	1 (2.0)
**Rash**	3 (5.9)	0 (0.0)
**Pancreatitis**	2 (3.9)	0 (0.0)
**Pneumonitis**	1 (2.0)	1 (2.0)
Other ^a^	4 (7.8)	2 (3.9)

a Oral ulcer/mucositis, Arthritis and Cardiac toxicity.

### Association of cytokines with irAEs

**Figure 1:**
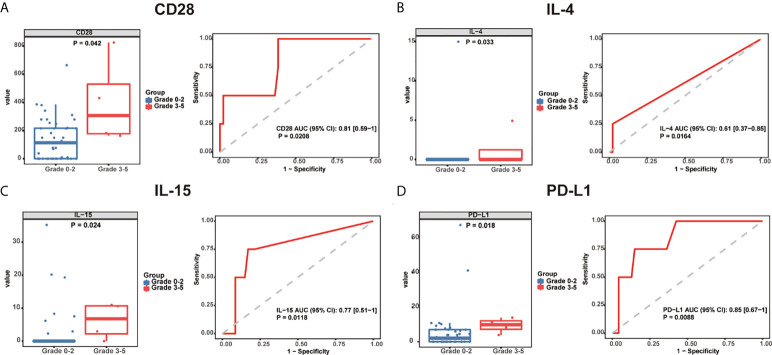
Baseline serum cytokine levels are significantly associated with irAE development and severity. Box plots (left) showing the distribution of serum cytokines **(A)** CD28, **(B)** IL-4, **(C)** IL-15, and **(D)** PD-L1 in grade 0-2 and 3-5 patients. ROC curve (right) analysis of sensitivity and specificity of serum cytokines **(A)** CD28, **(B)** IL-4, **(C)** IL-15, and **(D)** PD-L1 from baseline, to distinguish between grade 0-2 and 3-5 irAEs. The median of each group and P-value were calculated using the Mann-Whitney U test (P < 0.05). irAEs: immune-related adverse events, ROC: receiver operating characteristics.

To identify potential circulating biomarkers of efficacy and/or toxicity, serum samples were tested for the levels of a broad array of analytes. None of the baseline cytokine expressions were found to be associated with the incidence of irAEs. Then, we explored the baseline cytokine expression in patients with grade 0-2 and grade 3-5 irAEs, and found that the expression of CD28 [306.1 pg/mL, 95% confidence interval (CI): (162.7-823.6) vs. 113.5 pg/mL, 95% CI: (0-214.8), P = 0.042], IL-4 [0 pg/mL, 95% CI: (0-4.91) vs. 0 pg/mL, 95% CI: (0-0), P = 0.033], IL-15 [6.76 pg/mL, 95% CI: (0-10.98) vs. 0 pg/mL, 95% CI: (0-0), P = 0.024], and PD-L1 [9.82 pg/mL, 95% CI: (3.85-13.9) vs. 2.05 pg/mL, 95% CI: (0-4.46), P = 0.018] was significantly elevated in patients with grade 3-5 irAEs compared to those with grade 0-2 irAEs (**Figure 1**).

### Cytokine profiles in organ-specific irAEs

**Figure 2:**
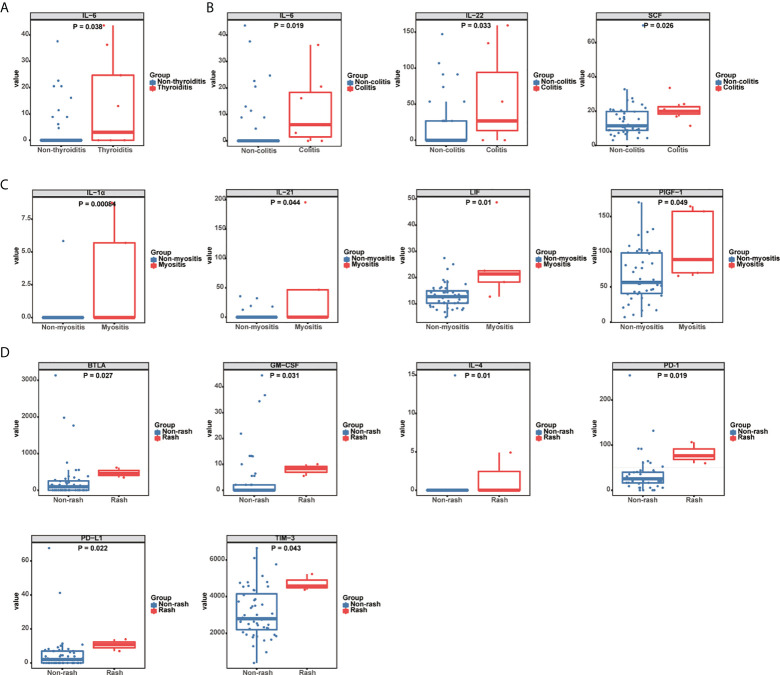
Association between serum cytokine levels and organ-specific irAEs. **(A)** Box plots representing serum IL-6 levels (pg/mL) in thyroiditis (n = 9) and non-thyroiditis (n = 42) patients. **(B)** Box plots representing serum IL-6, IL-22, and SCF levels (pg/mL) in colitis (n = 7) and non-colitis (n = 44) patients. **(C)** Box plots representing serum IL-1α, IL-21, LIF, and PIGF-1 levels (pg/mL) in myositis (n = 5) and non-myositis (n = 46) patients. **(D)** Box plots representing serum BTLA, GM-CSF, IL-4, TIM-3, PD-L1, and PD-1 levels (pg/mL) in patients with (n = 3) and without rash (n = 48). The median of each group and P-value were calculated using the Mann-Whitney U test (P < 0.05).

To explore biomarkers that could predict organ-specific irAEs in GI cancer patients treated with ICIs, we evaluated the cytokine profiles in various organ-specific irAEs. The results showed that enhanced IL-6 levels could predict the occurrence of thyroiditis. Compared to non-thyroiditis patients, basal IL-6 levels were significantly higher in thyroiditis patients [3.02 pg/mL, 95% CI: (0-36.24) vs. 0 pg/mL, 95% CI: (0-0), P = 0.038] (**Figure 2A**, **Supplementary Figure 1**). Among the predictive markers for colitis, the levels of IL-6 [6.12 pg/mL, 95% CI: (0-20.5) vs. 0 pg/mL, 95% CI: (0-0), P = 0.019], IL-22 [26.77 pg/mL, 95% CI: (0-134.5) vs. 0 pg/mL, 95% CI: (0-0), P = 0.033], and SCF [19.86 pg/mL, 95% CI: (16.93-24.16) vs. 11.47 pg/mL, 95% CI: (9.6-16.04), P = 0.026] were higher in patients with colitis compared to those without colitis (**Figure 2B**, **Supplementary Figure 1**). The incidence of myositis was associated with increased baseline levels of IL-1α [0 pg/mL, 95% CI: (0-8.75) vs. 0 pg/mL, 95% CI: (0-0), P = 0.00084], IL-21 [0 pg/mL, 95% CI: (0-195.4) vs. 0 pg/mL, 95% CI: (0-0), P = 0.044], LIF [21.39 pg/mL, 95% CI: (12.72-48.7) vs. 12.72 pg/mL, 95% CI: (10.51-14.33), P = 0.01], and PIGF-1 [88.76 pg/mL, 95% CI: (65.6-164.3) vs. 56.33 pg/mL, 95% CI: (46.8-77.37), P = 0.049] (**Figure 2C**, **Supplementary Figure 1**). We also investigated the prediction role of serum cytokines in ICI-related hepatitis, but found no significant difference between patients with and without hepatitis. Moreover, we evaluated the role of serum cytokines as predictive biomarkers for rash. The baseline levels of BTLA [460.6 pg/mL, 95% CI: (346.5-617.2) vs. 95.3 pg/mL, 95% CI: (0-145.2), P = 0.027], GM-CSF [8.35 pg/mL, 95% CI: (5.54-10.04) vs. 0 pg/mL, 95% CI: (0-0), P = 0.031], IL-4 [0 pg/mL, 95% CI: (0-4.91) vs. 0 pg/mL, 95% CI: (0-0), P = 0.01], PD-1 [76.42 pg/mL, 95% CI: (59.8-106.5) vs. 25.27 pg/mL, 95% CI: (20.0-26.2), P = 0.019], PD-L1 [10.77 pg/mL, 95% CI: (6.95-13.9) vs. 2.05 pg/mL, 95% CI: (0-4.16), P = 0.022], and TIM-3 [4577 pg/mL, 95% CI: (4380-5228) vs. 2789 pg/mL, 95% CI: (2465-3504), P = 0.043], were higher in patients who developed a rash than in those who did not (**Figure 2D**, **Supplementary Figure 1**).

### Association of irAEs with ICI response and prognosis

**Figure 3:**
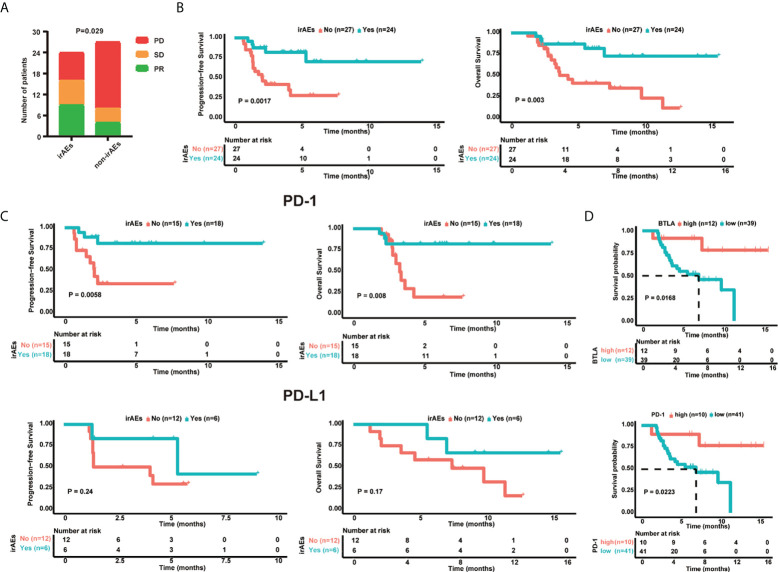
Baseline serum cytokine levels related to clinical outcomes. **(A)** Bar Chart showing the response rate in patients with (n = 24) and without irAEs (n = 27). **(B)** Kaplan-Meier survival curve of PFS and OS in the irAE (n = 24) and non-irAE (n = 27) groups. **(C)** Kaplan-Meier survival curve of progression-free survival (PFS) and overall survival (OS) following anti-PD-L1 (n = 18) and anti-PD-1 (n = 33) treatment in irAE and non-irAE groups. **(D)** OS of gastrointestinal cancer patients based on BTLA and PD-1 levels. CR: complete response, PR: partial response, SD: stable disease, PD: progressive disease, PFS: progression-free survival, OS: overall survival. Kaplan-Meier survival curves were plotted for patients using the median cutoff. Statistical significance was determined using the log‐rank (Mantel-Cox) regression analysis, with the level of significance at P ≤ 0.05.

Firstly, the treatment response in patients with and without irAEs, who received ICIs, was compared. The results showed that patients with irAEs had better tumor response (P = 0.029), with higher percentages of PR (37.5% vs.14.8%) and SD (29.2% vs. 14.8%), and a lower percentage of PD (33.3% vs.70.4%) (**Figure 3A**, **Table 1**). Patients with irAEs also had higher PFS (median survival: undefined vs. 2.1 months, P = 0.002) and OS (median survival: undefined vs. 4.3 months, P = 0.003) (**Figure 3B**). Then, we investigated the prognosis of patients who received anti-PD-1 and anti-PD-L1 inhibitors separately. Patients who received anti-PD-1 inhibitors and developed irAEs had higher PFS (median survival: undefined vs. 2.1 months, P = 0.006) and OS (median survival: undefined vs. 3.3 months, P = 0.008) (**Figure 3C**). In patients who received anti-PD-L1 inhibitors, there were no significant differences in PFS (median survival: 5.37 vs. 2.73 months, P = 0.24) and OS (median survival: undefined vs. 7.4 months, P = 0.17). Furthermore, we explored the prognostic value of baseline cytokines and found that patients with elevated BTLA (HR: 5.41; 95% CI: 1.19-24.58; log-rank P = 0.0168) or PD-1 concentrations (HR: 5.127; 95% CI: 1.12-23.46; log-rank P = 0.0223) had higher OS (**Figure 3D**). However, none of the cytokines were associated with significant differences in PFS.

## Discussion

PD-1/PD-L1 axis blockade has reshaped the landscape of tumor therapy, and has yielded unprecedented clinical success in multiple advanced cancer types, including GI cancers (16, 17). The use of ICIs is rising exponentially, with the development of novel ICIs and expanding clinical trials for immunotherapy (18). ICIs block key immunoregulatory pathways, thereby increasing antitumor immunity, but can also cause irAEs. In this study, we investigated 51 GI cancer patients who received anti-PD-1/PD-L1 monotherapy, and found a 47.1% incidence of irAEs, including 7.8% grade 3-5 irAEs. This was similar to a number of previous studies, which have reported an incidence of 36.6-85% for all grades and 12.6-26% for grades 3-5 in patients receiving monotherapy (19–21). The incidence could approach 95% when combination therapies were used (22, 23). In addition to the high morbidity of irAEs, a recent meta-analysis reported a fatality rate of 1.3%, and as many as one third of the patients were forced to terminate promising therapies due to toxicity (24). Therefore, early recognition of severe irAEs is critical in clinical practice for appropriate management of toxicities and improving medical safety.

We explored baseline cytokine expression in GI cancer patients, and found that CD28, IL-4, IL-15 and PD-L1 expression was significantly elevated in patients with grade 3-5 irAEs compared to patients with grade 0-2 irAEs. Evidence suggests that the CD28/CTLA-4 and PD-1/PD-L1 family play fundamental roles in the regulation of strength, quality, and/or duration of the lymphocytic antigen receptor signal, and in the development and maintenance of immune tolerance (25). IL-4 is a cytokine that induces proliferation/differentiation of T cells and stimulates B cell activation. It is also a key regulator of humoral and adaptive immunity (26). IL-15 induces proliferation of natural killer cells and is characterized as a T cell growth factor (27). In summary, cytokines possess potent proinflammatory properties, including stimulation of immune cell proliferation, differentiation, and effector functions; therefore, enhanced CD28, IL-4, IL-15 and PD-L1 levels might indicate uncontrolled severe irAEs.

IrAEs can affect a variety of organs, and may be associated with a serious decline in organ function and quality of life (28, 29). Therefore, organ-specific irAEs are more meaningful and require early detection and appropriate management (30). However, the mechanisms of organ-specific irAEs remains unclear. Previous studies have suggested that excessive release of cytokines activates specific immune components, e.g., GI irAEs are associated with IL-17 (10), and cutaneous with IL-6 and IL-10 (31), while immune-related pneumonia is associated with CD74 (32). However, for different tumor types, the predictive markers remain discrepant (4, 33–35). To determine predictive markers for GI cancer patients, we explored relationships between specific cytokines and organ-specific irAEs. In our study, IL-6 levels were higher in patients with thyroiditis and colitis, while IL-22 and SCF levels were higher in patients with colitis. Increased IL-1α, IL-21, LIF, and PlGF-1 levels were significantly associated with the incidence of myositis, and the serum levels of six cytokines (BTLA, GM-CSF, IL-4, PD-1, PD-L1 and TIM-3) were higher in patients who developed a rash. These cytokines, produced by immune or tumor cells, possess immunoregulatory functions in many infectious and inflammatory disorders (36–39). Some of the cytokines exert proinflammatory effects (36–38), while some compromise the immunity in tumor-bearing individuals (39–41). Although the exact mechanism remains unclear, it has been suggested that pro- and anti-inflammatory factors interact in patients with adverse reactions.

An association between the occurrence of irAEs and ICI clinical efficacy has been reported (42, 43), but the results remain controversial (44, 45). In this study, we found that patients with irAEs had better tumor response and prognosis. The mechanisms underlying the association between irAEs and survival benefits are not completely understood. Antigen mimicry theory is considered one of the most promising hypotheses. Preclinical data have identified multiple epitopes that are shared between tumor and normal cells (46, 47). The release of shared antigens by ICI therapy might result in an overlap of immune reactions toward tumors and normal tissues (48), so the occurrence of irAEs may predict better treatment responses. We also demonstrated the distinct prognostic value of irAEs for patients who received anti-PD-1 or anti-PD-L1 inhibitors. Similar results have also been reported previously, i.e., that the association between irAEs and favorable survival outcomes was significant for patients who received PD-1 inhibitors, but not CTLA-4 inhibitors (49). The reason for this difference remains to be explored.

There were several limitations of this study. Although serum cytokines are convenient and cost-effective as predictive markers for safety, validation studies with larger samples are required to prove their significance. Besides, cytokine levels change dynamically with ICIs, so blood specimens for correlative studies were planned at baseline and at several weeks. Moreover, the molecular mechanism for serum cytokines to predict irAEs was still unclear, which should be a direction for further study in the future.

In conclusion, serological proteins are promising markers that predict severe immune-related toxicity and prognosis in GI cancer patients treated with anti-PD-1/anti-PD-L1 immunotherapy. Organ-specific irAEs have various cytokine profiles. Although larger, multi-institutional cohorts are needed for further validation, these findings have important clinical implications for understanding and managing severe, potentially life-threatening irAEs.

## Data availability statement

The original contributions presented in the study are included in the article/**Supplementary Material**. Further inquiries can be directed to the corresponding authors.

## Ethics statement

The studies involving human participants were reviewed and approved by Clinical Research Ethics Committee of Peking University Cancer Hospital and Institute Consent. The patients/participants provided their written informed consent to participate in this study.

## Author contributions

ZL, HZ and LS designed the study. JG, JiaL, XZ, XW, ZP, CQ, ZW, JieL and YanL contributed to patient enrolment and data acquisition. XJ, YJW, NZ and MG analyzed and interpreted data. YNW,JZ and YunL performed bioinformatics analysis. YNW and JZ did experiments and wrote the manuscript. All authors read and approved the final manuscript.

## Funding

This study was supported by the Clinical Medicine Plus X-Young Scholars Project of Peking University (PKU2021LCXQ030), the Digestive Medical Coordinated Development Center of Beijing Hospitals Authority (No. XXT19), Beijing Hospitals Authority Youth Programme (QML20191102), Natural Science Foundation of Shanghai (20YF1408900) and Beijing CSCO Clinical Oncology Research Foundation (Y2019Genecast-074).

## Acknowledgments

We thank textcheck (http://www.textcheck.com) for providing linguistic assistance during the preparation of this manuscript.

## Conflict of interest

The authors declare that the research was conducted in the absence of any commercial or financial relationships that could be construed as a potential conflict of interest.

## Publisher’s note

All claims expressed in this article are solely those of the authors and do not necessarily represent those of their affiliated organizations, or those of the publisher, the editors and the reviewers. Any product that may be evaluated in this article, or claim that may be made by its manufacturer, is not guaranteed or endorsed by the publisher.
