# Supplementary material for: Serological biomarkers predict immune-related adverse events and clinical benefit in patients with advanced gastrointestinal cancers
Source: Front Immunol. 2022 Sep 8;13:987568. doi: 10.3389/fimmu.2022.987568 (PMC9492966; doi:10.3389/fimmu.2022.987568)
Supplement: Supplementary file 1 [file DataSheet_1.docx]

Supplementary Material


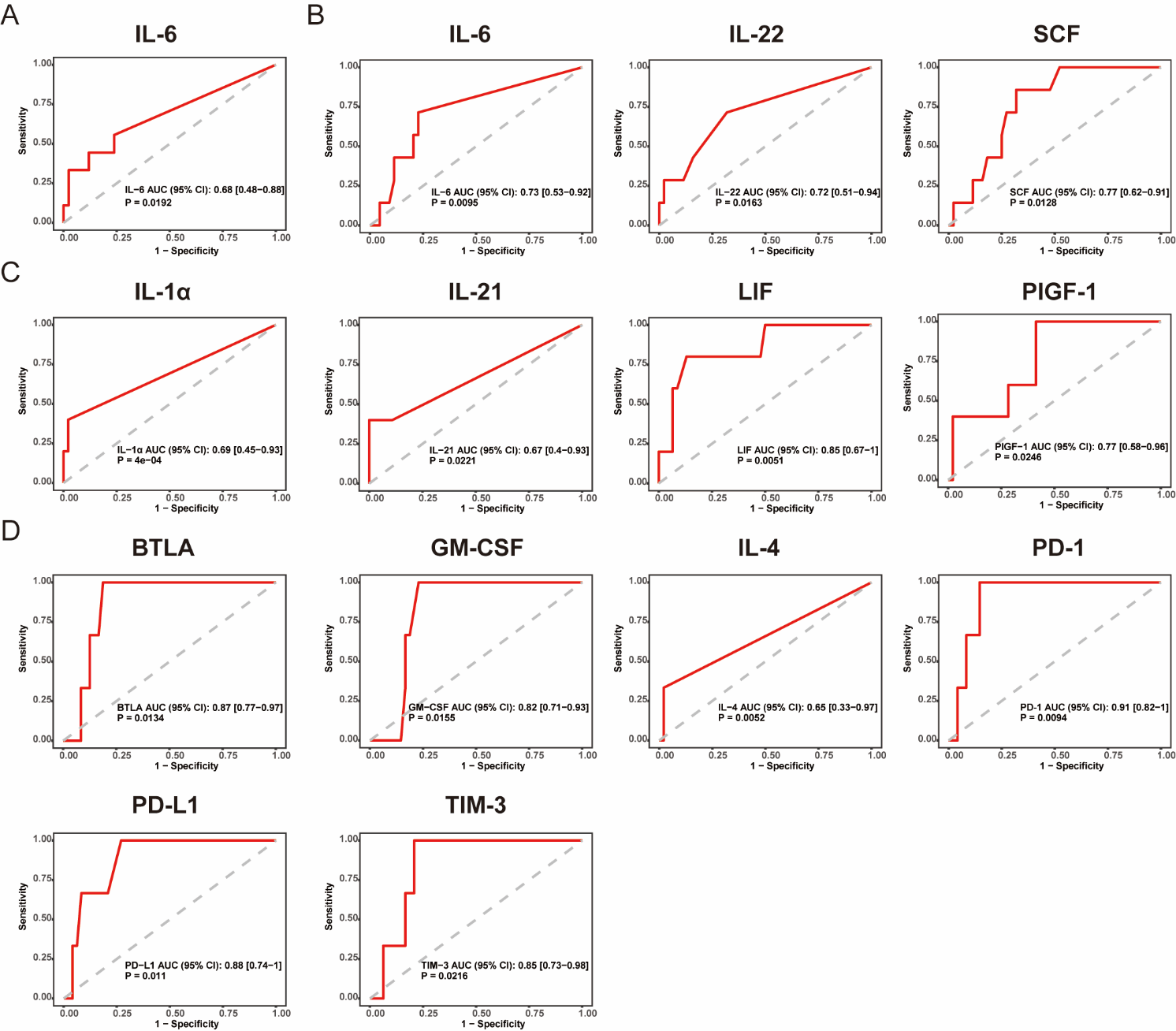


**Supplementary Figure 1.** **ROC curve analysis for the prediction of organ-specific irAEs based on a specific set of cytokines.** (A) ROC curves and corresponding AUC values of IL-6 in thyroiditis patients. (B) ROC curves for IL-6, IL-22, and SCF as biomarkers for the risk of colitis. (C) ROC curves for IL-12, IL-21, LIF, and PIGF-1 as biomarkers for the risk of myositis. (D) ROC curves for BTLA, GM-CSF, IL-4, TIM-3, PD-L1, and PD-1 as biomarkers for the risk of rash. ROC: receiver operating characteristics; AUC: area under the curve.
